# Role of Toll-like receptor 4 signaling in mast cell-mediated migraine pain pathway

**DOI:** 10.1177/1744806919867842

**Published:** 2019-08-08

**Authors:** Roshni Ramachandran, Zhenping Wang, Christian Saavedra, Anna DiNardo, Maripat Corr, Susan B Powell, Tony L Yaksh

**Affiliations:** 1 Department of Anesthesiology, University of California San Diego, La Jolla CA, USA; 2 Department of Dermatology, University of California San Diego, La Jolla, CA, USA; 3 Division of Rheumatology, Allergy, and Immunology, University of California San Diego, La Jolla, CA, USA; 4 Department of Psychiatry, University of California San Diego, La Jolla, CA, USA

**Keywords:** Migraine, TLR4, mast cell, compound 48/80, MyD88

## Abstract

Degranulation of meningeal mast cells leading to the sensitization of trigeminal vascular afferent processing is believed to be one of the mechanisms underlying the migraine pain pathway. Recent work suggests that Toll-like receptor 4 (TLR4) may be involved in signaling states of central sensitization. Using a murine model of light aversion produced by compound 48/80 (2 mg/kg, intraperitoneal) mast cell degranulation, employed as a surrogate marker for photophobia observed in migraineurs, we examined the role of TLR4 in migraine-like behavior and neuronal activation. Using a two-chambered light/dark box, we found that compound 48/80 administration in male and female C57Bl/6 mice produced light aversion lasting up to 2 h, and that pre-treatment with sumatriptan (1 mg/kg, i.p.) reliably prevented this effect. Genetic deletion and pharmacological blockade of TLR4 with TAK-242 (3 mg/kg, i.p.) reversed the light aversive effects of compound 48/80 in males but not in females. Assessing the downstream signaling pathway in mutant mice, we found that the TLR4-mediated, light aversion was dependent upon myeloid differentiation primary response gene 88 but not Toll-interleukin-1 receptor domain-containing adapter-inducing interferon-β signaling. In separate groups, male mice sacrificed at 10 min following compound 48/80 revealed a significant increase in the incidence of evoked p-extracellular signal–regulated kinases (+) neurons in the nucleus caudalis of wild type but not *Tlr4^−/−^* mice or in mice pre-treated with sumatriptan. This study thus provides the first evidence for involvement of TLR4 signaling through MyD88 in initiating and maintaining migraine-like behavior and nucleus caudalis neuronal activation in the mouse.

## Introduction

Migraine is characterized by episodic pain referred to the head and accompanied by phenomena such as photophobia.^1,2^ Factors such as chronic stress or events such as cortical spreading depressions (CSDs) can generate a state of ‘sterile inflammation’ in the meninges, resulting in the sensitization and activation of meningeal nociceptors.^3–6^ Mast cells densely populate the meninges. They lie in close vicinity to meningeal afferent fibers and vasculature.^7^ Accordingly, activation of mast cells is considered to be a key link in mediating neuro-immune interactions that lead to a local sterile inflammation of the meninges. Thus, activation of meningeal mast cells by compound 48/80 promotes sensitization and activation of meningeal afferents, which subsequently release a variety of neuroactive products, including substance P and calcitonin gene-related peptide (CGRP). These agents can induce vasodilation and plasma extravasation which lead to activation of the primary trigeminovascular afferent and then the second-order neurons in the nucleus caudalis.^8–10^ Activation of neuraxial neurons by traffic in small afferent fibers is robustly enhanced by neuraxial neuro-immune and neuro-glial interactions, and these systems may play a role in the development of persistent pain states.^11–13^ In addition, plasma and cerebrospinal fluid levels of afferent peptides (CGRP) and pro-inflammatory cytokines such as tumor necrosis factor (TNF) and interleukin-1β (IL-1β) are enhanced during migraine attacks,^14–16^ emphasizing the contribution of neuro-immune interactions in migraine pathogenesis. The role of neuro-immune cascades in the expression of the neuro-inflammation in the migraine phenotype, however, remains unclear.

Toll-like receptor 4 is a part of the innate immune system and is expressed on neurons, microglia, and astrocytes.^17,18^ Toll-like receptor 4 (TLR4) responds to diverse pathogen-associated molecular patterns such as lipopolysaccharide (LPS).^19^ Following injury or cellular stress, several endogenous molecules referred to as danger-associated molecular patterns, such as high mobility group box 1 (HMGB1), heat shock proteins (HSP), and fibronectin and hyaluronic acid, can also activate TLR4 signaling.^20,21^ Of particular relevance, HMGB1 is released in the cerebral cortex following CSD.^22^ Furthermore, upregulation of HSP70 and TLR4 in trigeminal ganglion neurons following tooth pulp inflammation has been reported,^23^ suggesting that endogenous processes such as CSD and inflammation that are linked to migraine can result in TLR4 activation. Interestingly, contribution of TLR4 pathway has been suggested in inducing hyperalgesia in dural inflamed rats.^24^ TLR4 signals in parallel through two adaptor proteins: Myeloid differentiation primary response gene 88 (MyD88) and TIR-domain-containing adapter-inducing interferon-β (TRIF). The balance of activation of these two pathways determines the downstream signaling, mediating the release of cytokines such as TNF, or interferon β (IFN-β),^19,25^ previously associated with migraine attacks.

These findings suggest a mechanistic hypothesis supporting the underlying role of neuro-immune signaling, in particular, through TLR4 signaling in the migraine pain phenotype. In this study, we characterized the role of TLR4 signaling in the mouse using the light aversion model induced by meningeal mast cell degranulation using a systemic mast cell degranulator (compound 48/80).

## Methods

### Animals

The protocol was approved by the Institutional Animal Care and Use Committee at the University of California, San Diego. Mice were housed up to four per standard cage while maintaining a 12:12-h light/dark cycle, with food and water available ad libitum. All of the procedures and testing were conducted during the light portion of the cycle. Male and female wild-type C57Bl/6 mice were purchased from Harlan (Indianapolis, IN). The *Tlr4^−/−^* and *Myd88^−/−^* mice were a gift from Dr S Akira (Osaka University, Japan) and were backcrossed for 10 generations onto the C57Bl/6 background. *Ticam1^lps^*^2^ mice were a gift from Dr B Beutler (University of Texas Southwestern, Tx) and were directly generated on the C57Bl/6 background. *Myd88^−/−^* mice and *Ticam1^lp2^* mice were intercrossed to generate *Ticam 1^lps2^/Myd88^−/−^* mice. Mast cell deficient mice *Kit^wsh−/−^* on C57Bl/6 background were obtained from Dr Besmer (NY) and bred at UCSD.

### Drugs and drug delivery

All drugs were prepared from stock solutions. Compound 48/80 (Sigma-Aldrich, St Louis, MO) at a dose of 2 mg/kg was administered intraperitoneally. Sumatriptan (Sigma-Aldrich, St Louis, MO), Cromolyn (Sigma-Aldrich, St Louis, MO), and TAK-242 (Epigen Biosciences Inc, San Diego, CA) at doses of 1 mg/kg, 10 mg/kg, and 3 mg/kg, respectively, were administered intraperitoneally. TAK-242 was dissolved in 5% dimethyl sulfoxide and 5% Tween80 and brought to the final volume using 0.9% saline. Stability of the formulated TAK-242 was confirmed by high-performance liquid chromatography/mass spectrometry (M + H 362.8). All solutions were stored at 4°C and brought to room temperature (RT) prior to use.

### Light aversive behavior

All behavioral tests were conducted at fixed times (9:00 a.m.–5:00 p.m.). Light aversive behavior was performed using a light and dark box. This test system is composed of two equally sized compartments A and B (each measuring 90 × 90 × 165 cm), animals can move freely from one to the other through a small portal. One of the chambers was illuminated with light (7000 lux) and the other chamber painted dark. During the testing period, the box is covered with an opaque lid. The animals were acclimatized for 20 min in the chamber one day before testing. On the day of testing, baseline values were obtained prior to the injection with saline or the drug i.p. Each testing period lasted for 15 min. Following the administration of saline or compound 48/80, animals were tested at 15 min, 1 h, 2 h, and 4 h. Time spent in each chamber was measured by the animal’s obscuration of the light path of three red LED lights mounted close to the floor in each chamber. Time in each chamber was collected automatically and placed in spread sheets. Percentage of time spent in the light chamber was then calculated and plotted on a graph.

### Open-field assay for anxiety

The video-tracker (VT) consisted of four adjacent white plastic enclosures (41 × 41 × 34 cm) surrounded by a white plastic curtain. Each mouse was tested individually in a separate enclosure. A video camera, mounted 158 cm above the enclosures, provided the signal for the Ethovision 3.1 software (Noldus Information Technology, Leesburg, VA). The number of entries and time spent in the center area (25 × 25 cm) and overall distance traveled were computed by Ethovision 3.1.

One hour after i.p administration of compound 48/80 or vehicle or pre-treatment with sumatriptan followed by compound 48/80, each mouse was placed in the bottom left hand corner of each enclosure at the start of the test session. The movements of the mice were tracked for 30 min, with data being stored in 6 or 12, 5-min blocks, respectively. The amount of locomotor activity was measured by the distance traveled, i.e., tracing the consecutive locations of the animal using the highest resolution of the VT and calculating the distance between them. Three different parameters were measured: (1) number of entries to the center, (2) time spent in the center, and (3) distance traveled.

### p-Extracellular signal–regulated kinases immunostaining

To assess p-extracellular signal–regulated kinases (ERK) (a marker of dorsal horn neuron activation), mice were anesthetized and perfused with 0.9% saline followed by 4% paraformaldehyde, and the brainstem was harvested at 15 min following i.p saline or compound 48/80 administration. For pre-treatment studies, sumatriptan (1 mg/kg) or TAK-242 (3 mg/kg) was administered i.p. 45 min or 3 h prior to i.p. compound 48/80 administration following which mice were perfused and brainstems were harvested at 15 min. Tissues were post fixed and cryoprotected in sucrose. Cross-sections of trigeminal nucleus caudalis (TNC) were cut, with each section having a thickness of 30 μm. Every fourth section was subjected to p-ERK immunostaining, rinsed in PBS (phosphate-buffered saline), then permeabilized in a solution of 0.3% Triton X-100 (Sigma Chemicals, St. Louis, MO) in PBS (PBS-TX). Nonspecific binding was blocked with 10% normal goat serum (Vector Laboratories, Inc., Burlingame, CA) diluted in PBS and sections were incubated for 1 h at RT. The p-ERK polyclonal antibody raised in rabbit (Calbiochem) was diluted to a concentration of 1:1000 in PBS-TX and incubated overnight at RT. After rinsing in PBS, sections were incubated for 1 h at RT with biotinylated goat anti-rabbit IgG (Vector Laboratories) diluted in PBS. Tissue sections were then rinsed in PBS and incubated for 1 h at RT in Avidin-Biotin Complex-Horse Radish Peroxidase complex (Vector Laboratories) per manufacturer’s directions. After further rinsing of tissue in PBS, the sections were incubated in 3,3′ diaminobenzidine chromogen until a precipitate was visible on the positive sites. A final rinse in water was performed to stop the chromogenic reaction. Tissue sections were then slide mounted, dehydrated, and cover slipped with Dibutylphthalate Polystyrene Xylene non-aqueous mounting media (Electron Microscopy Sciences, Hatfield, PA) for microscopic evaluation. Total number of p-ERK-positive cells stained in each section was counted rostrocaudally. The Vi/Vc region was defined as the distance between 0 and 0.6 mm, Vc region between 0.6 mm and 1.5 mm and C1/C2 above 1.5 mm and 1.8 mm from the obex. There were a minimum of two to a maximum of three sections taken from each division depending upon the quality of the sections. An observer blinded to the treatment performed counting of the p-ERK-positive cells in the region of interest. The number of p-ERK-positive cells was counted bilaterally within these three regions (Vi/Vc, Vc and C1/C2). The mean number of p-ERK-positive cells was calculated from the total number of cells/3 sections between each division from each animal.

### Mast cell culture and degranulation assay

To examine the role of TLR4 in 48/80 evoked mast cell degranulation, primary murine mast cells were generated from C57BL/6 mouse bone marrow and cultured in Roswell Park Memorial Institute 1640 medium (Invitrogen) supplemented with 10% heat-inactivated fetal bovine serum (Thermo Fisher Scientific, Chicago, IL), 25 mM HEPES (pH 7.4), 4 mM L-glutamine, 0.1 mM nonessential amino acids, 1 mM sodium pyruvate, 50 mM 2-ME, 100 IU/ml penicillin, and 100 mg/ml streptomycin. Mast cells derived from bone marrow cells were cultured with recombinant murine SCF (stem cell factor, 20 ng/ml) and IL-3 (1 ng/ml) (Peprotech, Rock Hill, NJ) to allow for in vitro differentiation. After four weeks, the mast cells were fully differentiated, as confirmed by the expression of CD117 (c-Kit) and FcεRI. Cell maturation was confirmed by metachromatic staining with toluidine blue. The purity of mast cells was greater than 98%.

To determine if mast cell degranulation was affected by TLR4 blockade, mast cells were treated with compound 48/80 and a TLR4 antagonist (TAK-242). Degranulation was assessed by measuring the activity of β-hexosaminidase in the supernatants of 1 × 10^5^ MCs in 200 µL Tyrode’s buffer incubated for 1 h with TAK-242 (50 nM, 500 nM, and 1000 nM) before the addition of 10 μg/mL compound 48/80 (Sigma-Aldrich, St Louis, MO). For each sample assayed, supernatant aliquots (20 μL) were mixed with substrate solution (100 μL) which consisted of 10 mM 4-methylumbelliferyl-2-acetamide-2-deoxy-b-D-glucopyranoside (Calbiochem; EMD Millipore, Billerica, MA) in 0.1 M sodium citrate buffer (pH 4.5) and were incubated for 1 h at 37°C. The reaction mixtures were excited at 365 nm and measured at 460 nm in a fluorescence plate reader (Gemini EM microplate spectrofluorometric; Molecular Devices, Sunnyvale, CA). To determine the total cellular content of this enzyme, an equivalent number of mast cells were lysed with 1% Triton X-100. Release of β-hexosaminidase was calculated as the percentage of the total enzyme content.

### Statistical analysis

All values are presented as mean ± standard error of the mean (SEM). Calculation of group size was based on a power analysis where the group mean and standard deviation (SD) for light aversive behavior in photophobic mice are nominally 21.76% ± 8.084% (of time spent in light) (N = 6). Maximum percentage of time spent in light by a normal mouse is 52.3 ± 10.39. Assuming a 30% reversal is significant, we can assume α = P < 0.05 and a statistical power (1−β) of 80% for sample sizes of 6. Previous work has confirmed robustness of data for such group sizes. Statistical analysis protocols for each data set were based on the results of testing the null hypothesis of normality (Kolmogorov–Smirnov test) and homogeneity of variances (Bartlett’s test). Graphics of data sets for which the null hypotheses were not rejected present the data as means ± SD and employed parametric statistics (one-way or two-way repeated measures analysis of variance (ANOVA) analysis with post hoc comparisons being made using the Dunnett’s test). Adjusted P values for all the post hoc tests performed are presented in Supplemental Table 1. Data in Figure 7 are analyzed using ordinary one-way ANOVA. Differences reaching the level of P < 0.05 were considered to be significant. Prism (GraphPad Prism software Inc., version 5.0, San Diego, CA, USA) was used for statistical analysis.

## Results

### Compound 48/80-induced light aversion in C57Bl/6 WT

**Figure 1.**
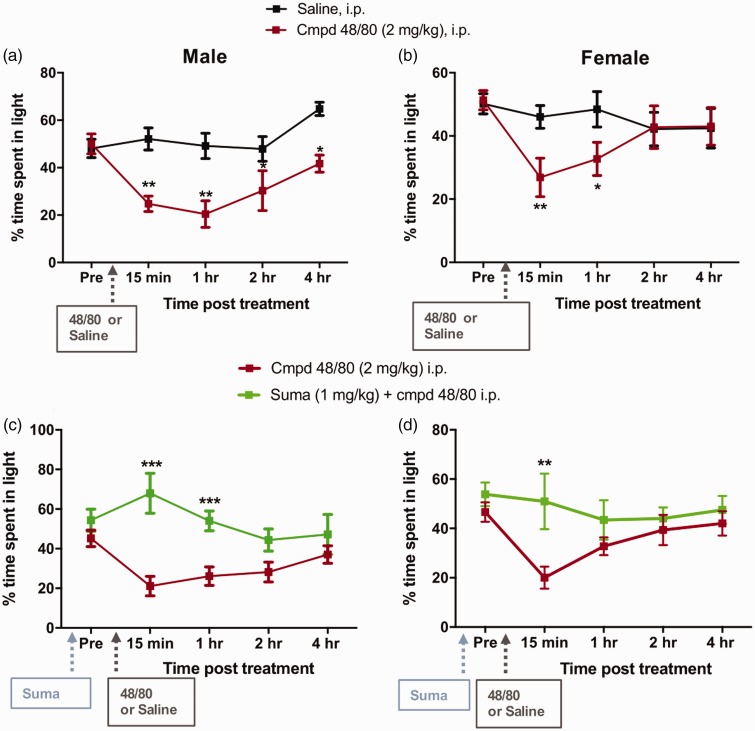
Percentage of time spent in the light chamber: Intraperitoneal compound 48/80 (2 mg/kg)-induced light aversion starting at 15 min that lasted up to 2 h in males (a) and approximately 1 h in females (b) as compared to the saline-treated groups. Data are expressed as mean ± SEM, N = 6–8. *P < 0.05, **P < 0.01 as compared to vehicle. Pre-treatment with i.p. sumatriptan (1 mg/kg) significantly attenuated the light aversive effects of compound 48/80 in males (c) and in females (d). Data are expressed as mean ± SEM, N = 6–8. **P < 0.01, ***P < 0.001 as compared to compound 48/80-treated group, N = 6–8.

In untreated, C57Bl/6 WT mice following the adaptation period, the time spent in the light/dark chamber over a 15 min exposure was approximately 55%/45%, respectively. In males and females i.p. compound 48/80 (2 mg/kg) produced a significant increase in the time spent in the dark chamber starting at 15 min (P < 0.01) with a long-lasting effect observed up to 2 h by males as compared to the control group that received saline (P < 0.05) (Figure 1(a)). A complete reversal to baseline was observed by 4 h when compared to the baseline values. In females, a similar light aversion was observed with i.p. compound 48/80, though the effects lasted up to 1 h (P < 0.05) with a complete reversal by 2 h (Figure 1(b)).

### Sumatriptan attenuated compound 48/80 evoked light aversion

To validate the model for migraine-like behavior, we pre-treated the animals with sumatriptan (1 mg/kg, i.p.), 45 min prior to i.p. compound 48/80. Pre-treatment with sumatriptan significantly attenuated compound 48/80-induced light aversive behavior both in males (P < 0.001) and in females (Figure 1(c) and (d)), showing that sumatriptan was effective in inhibiting avoidance to light following compound 48/80 administration.

### Light aversive behavior induced by compound 48/80 is not an anxiety related-behavior alone

**Figure 2.**
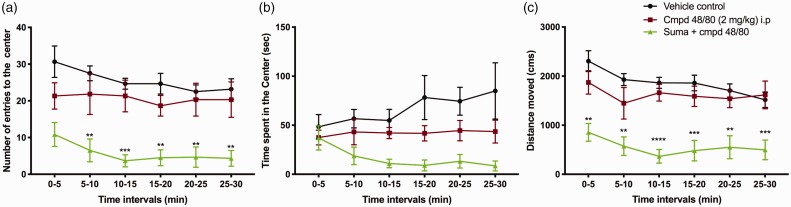
Examining effects over time for mice placed in an open field (anxiety-assay) and followed for the (a) number of entries into the center, (b) time spent in the center, and (c) distance moved. As indicated, C48/80 did not alter entries as compared to saline (e.g., did not increase anxiety) and sumatriptan resulted in a significant suppression of entries. Two-way ANOVA, post hoc Dunnett’s test: **P < 0.01; ***P<0.001 and ****P < 0.0001 compared to saline-treated group, N = 6.

To assess whether compound 48/80-induced light aversion was anxiety related, we tested the animals in an open-field assay. Since the light aversive behavior peaked at 1 h following systemic compound 48/80, we chose this time point for open-field assay where three parameters were observed: (1) time spent in the Center, (2) number of entries to the center, and (3) distance traveled. Interestingly, we did not observe a significant difference between the vehicle and the compound 48/80 treated group (Figure 2). Infact, we saw a significant reduction in the time spent in the center and the distance moved in the group treated with sumatriptan. This finding strongly suggests that the C48/80-evoked photophobia that’s reversed by sumatriptan in our model is a marker of migraine versus an anxiety phenotype.

### Compound 48/80-induced light aversion is prevented by genetic and pharmacologic blockade of TLR4 signaling in males but not in females

**Figure 3.**
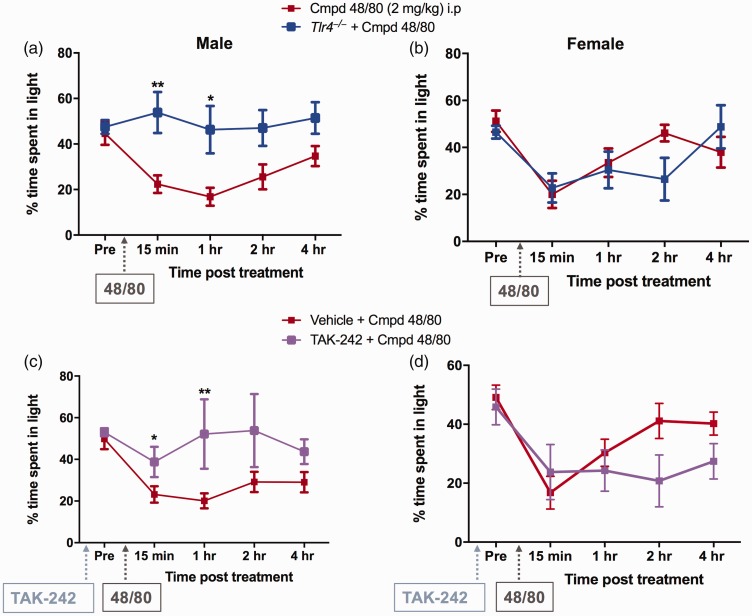
Genetic and pharmacological intervention of TLR4 attenuated compound 48/80-induced light avoidance in males but not in females: Percentage of time spent in the light chamber following i.p. compound 48/80 significantly increased in *Tlr4^−/−^* male mice (a). Compound 48/80-induced light aversiveness was not affected in *Tlr4^−/−^* female mice (b). Similarly, pre-treatment with TLR4 antagonist (TAK-242, 3 mg/kg) i.p. attenuated compound 48/80-induced light aversive behavior in males (c) with no effect seen in females (d). Data are expressed as mean ± SEM, N = 6–8. *P < 0.05 and **P < 0.01, as compared to compound 48/80 treated group, N = 6–8.

To determine the role of TLR4 in compound 48/80-induced sensitivity to light, we administered 48/80 in *Tlr4^−/−^* male and female mice. Compound 48/80 did not induce light avoidance behavior in males at any of the time points (Figure 3(a)). Surprisingly, female *Tlr4^−/−^* mice were no different than the WTs (Figure 3(b)), suggesting that TLR4 may not be critical to 48/80-mediated migraine-like behavior in females as in males.

To determine if pharmacological intervention of TLR4 showed similar results as those observed in *Tlr4^−/−^* mice, we pre-treated WT mice with TAK-242 (3 mg/kg) 3 h prior to compound 48/80 administration. This dose was chosen based on the previous in vivo studies that showed effectiveness in attenuating pain behavior.^26^ Interestingly, we observed similar responses as observed with *Tlr4^−/−^* animals. In males, TAK-242 pre-treatment significantly attenuated compound 48/80-induced light avoidance (Figure 3(c)), whereas females pre-treated with TAK-242 were no different as compared to the WT mice treated with compound 48/80 (Figure 3(d)). These data again suggest that compound 48/80-induced light aversive effects are TLR4 dependent in males but not in females.

### Compound 48/80-mediated TLR4 signaling is MyD88 dependent and TRIF independent

**Figure 4.**
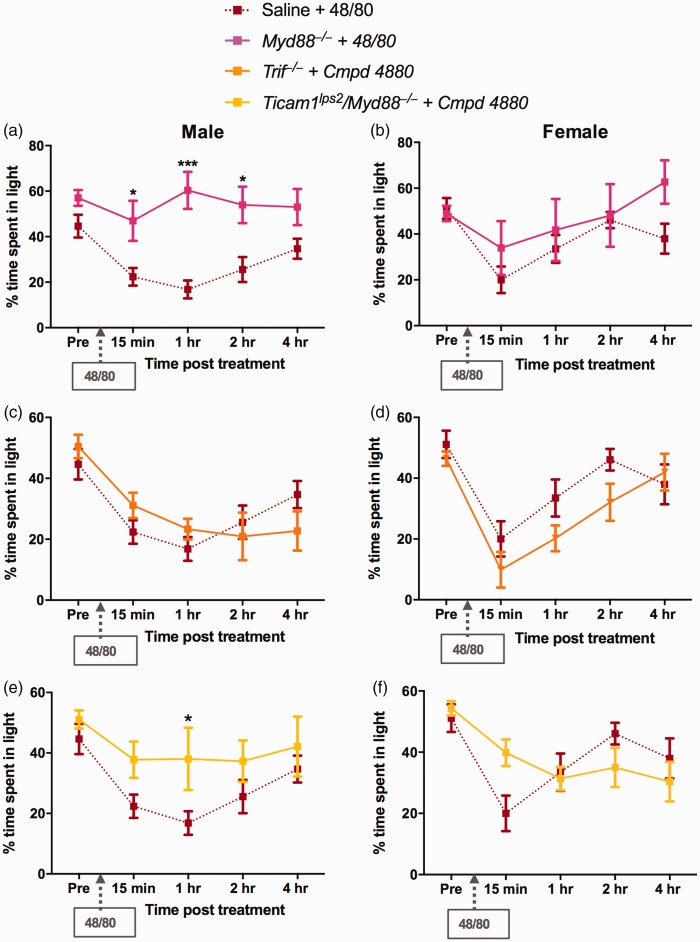
TLR4-mediated effects of compound 48/80 are MyD88 dependent and TRIF independent in males: Percentage of time spent in the light chamber following i.p. compound 48/80 significantly increased in *Myd88^−/−^* male mice (a) but not in females (b). Compound 48/80-induced light averseness were not affected in TRIF male (c), and female mice (d). Similarly, *Ticam1^lps^*2and *Myd88^−/−^* mutations attenuated the compound 48/80-induced behavioral effects in males (e) but not in females (f). These findings suggest that TLR4-mediated effects in this model are MyD88 dependent and TRIF independent in males. Data are expressed as mean ± SEM, N = 6–8. *P < 0.05 and ***P < 0.001 as compared to compound 48/80 treated group, N = 6–8.

As noted above, TLR4 acts through two major TLR signaling adapters, MyD88 and TRIF, to direct its downstream signaling pathways. Given that the deficiency and/or pharmacological blockade of TLR4 prevented the development of compound 48/80-induced light avoidance in male mice, we assessed the role of each adaptor protein in the compound 48/80-induced light aversion. Administration of 48/80 in *Myd88^−/−^* mice prevented the development of light aversive behavior in males (Figure 4(a)). However, consistent with the lack of effect of deleting TLR4 in females, compound 48/80 in *Myd88^−/−^* females did not show a reduction in light aversion (Figure 4(b)), suggesting no participation of TLR4-MyD88 signaling pathway in compound 48/80-induced light aversion in females. Administration of compound 48/80 in *Trif^−/−^* mice did not have any effect on the light aversive behavior in either males or females (Figure 4(c) and (d)), suggesting that compound 48/80-induced light aversion is MyD88 dependent and TRIF independent in males, whereas there is no involvement of TLR4/MyD88/TRIF signaling pathway in compound 48/80-induced light behavior in females. The above findings were also confirmed using dual deficiency of the adapter proteins MyD88 and TRIF. Male *Ticam1^lps2^/Myd88^−/−^* were protected from compound 48/80-induced effects, whereas females *Ticam1^lps2^/Myd88^−/−^* mice showed no difference in behavior as compared to WT that received compound 48/80 (Figure 4(e) and (f)).

### Compound 48/80-induced p-ERK expression in TNC of WT was prevented in Tlr4^−/−^ male mice

**Figure 5.**
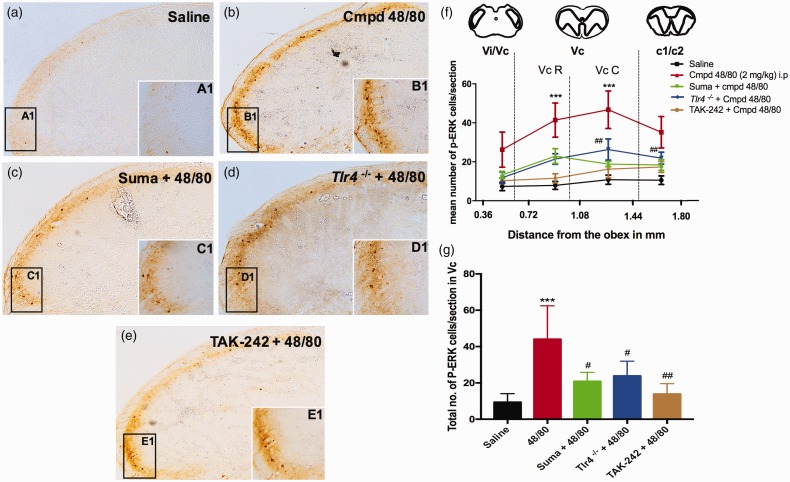
Lack of TLR4 receptor or pre-treatment with TLR4 antagonist (TAK-242) attenuated compound 48/80-evoked p-ERK expression in the trigeminal nucleus caudalis (TNC) in males: Representative images of TNC labeled with p-ERK-positive cells in the ventrolateral region following vehicle (a), compound 48/80 (b), Sumatriptan + compound 48/80 (c), *Tlr4^−/−^* + compound 48/80 (d), and TAK-242 + compound 48/80 (e) treatments. Line graph represents the rostrocaudal distribution of p-ERK-positive neurons in the TNC between 0.36 mm to 1.80 mm caudal to obex at 15 min after saline or compound 48/80 (f). Bar graph represents the total number of p-ERK-positive cells in the rostral and caudal Vc region (g), N = 4. ***P < 0.001 as compared to vehicle group. ^#^P <0.05 and ^##^P < 0.01 as compared to compound 48/80 treated group.

**Figure 6.**
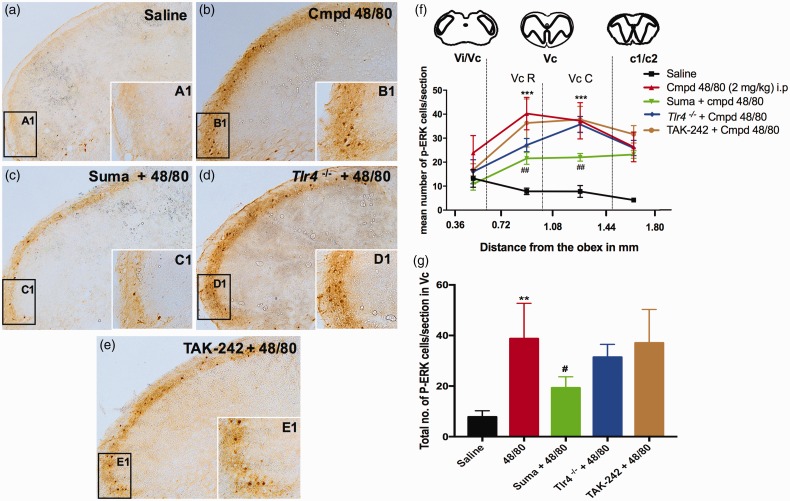
Lack of TLR4 receptor or pre-treatment with TLR4 antagonist (TAK-242) did not affect compound 48/80 evoked p-ERK expression in the trigeminal nucleus caudalis (TNC) in females: Representative images of TNC labeled with p-ERK-positive cells in the ventrolateral region following vehicle (a), compound 48/80 (b), Sumatriptan + compound 48/80 (c), *Tlr4^−/−^* + compound 48/80 (d), and TAK-242 + compound 48/80 (e) treatments. Line graph represents the rostrocaudal distribution of p-ERK-positive neurons in the TNC between 0.36 mm to 1.80 mm caudal to obex at 15 min after saline or compound 48/80 (f). Bar graph represents the total number of p-ERK-positive cells in the rostral and caudal Vc region (g), N = 4. ***P < 0.001 as compared to vehicle group. ^#^P <0.05 and ^##^P < 0.01 as compared to compound 48/80 treated group.

Intraperitoneal injection of compound 48/80 activated nocisponsive neurons as measured using p-ERK as a marker in the TNC of WT male and female mice. p-ERK-positive cells were counted in lamina I and II starting from 0.36 mm to 1.80 mm caudally to the obex. Quantitative analysis showed a significant increase in the number of p-ERK-positive neurons between 0.72 mm and 1.44 mm as compared to vehicle control animals both in males and in females. In contrast to the effects observed in WT mice, i.p 48/80 in *Tlr4^−/−^* mice prevented p-ERK expression in the TNC in males but not in females (Figures 5 and 6). Second, pre-treatment with the TLR4 antagonist TAK-242 (3 mg/kg, ip, 3 h prior), in a dose which we have previously shown to block direct activation of TLR4 signaling, significantly blocked compound 48/80-induced p-ERK expression in males alone.

### Systemic effects of compound 48/80

**Figure 7.**
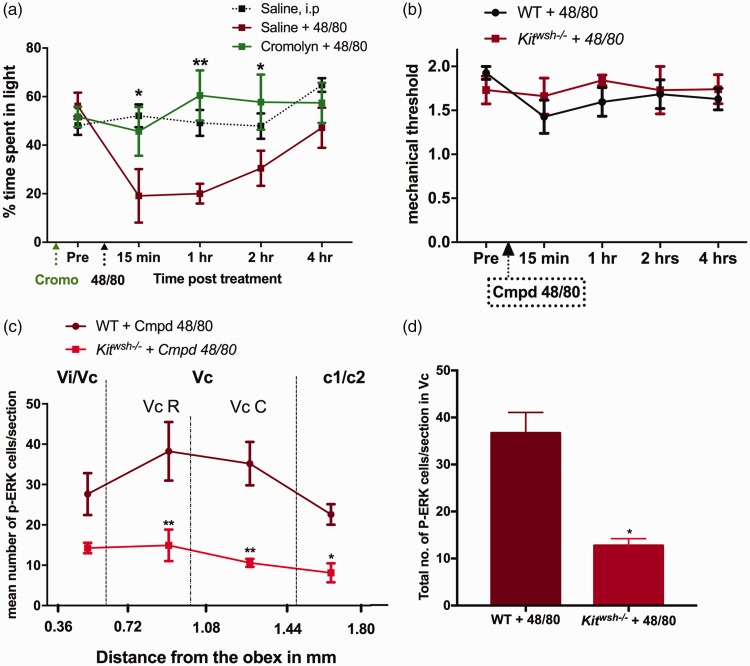
Systemic effects of compound 48/80: Pre-treatment with i.p. cromolyn (10 mg/kg) 30 min prior to 48/80 administration significantly attenuated the light aversive effects of compound 48/80, N = 6–8 (a). Intraperitoneal administration of compound 48/80 did not reduce hind paw mechanical thresholds in WT or mast cell deficient *Kit^wsh−/−^* male mice, N = 4 (b). Administration of 48/80 (i.p) did not activate p-ERK in the nucleus caudalis of *Kit^wsh−/−^* mice as compared to the wild type, N = 4. Data are expressed as mean ± SEM. *P < 0.05; **P < 0.01. WT: wild type.

In order to determine whether the 48/80-induced light aversive effects observed in this study were mast cell mediated, we pre-treated the male mice with Cromolyn sodium (10 mg/kg, i.p), a mast cell stabilizer, 30 min prior to compound 48/80 administration. We found that pre-treatment with cromolyn was effective in inhibiting 48/80-induced light aversion confirming that the behavioral effects of 48/80 observed in these studies were indeed mast cell degranulation dependent (Figure 7(a)). To further address the contribution of mast cells to the 48/80-induced photophobia, we utilized mast cell-deficient *Kit^wsh−/−^* mice. Unfortunately, we could not perform light aversive behavior in *Kit^wsh−/−^* mice, as they preferred spending 85% to 90% of their time in the dark chamber without any treatment. Furthermore, both the WT and *Kit^wsh−/−^* mice did not develop hind paw allodynia following systemic compound 48/80 (Figure 7(b)). However, we did look at the activation of p-ERK in WT and *Kit^wsh−/−^* following systemic compound 48/80. Compound 48/80 induced p-ERK activation in the nucleus caudalis of the WT mice but not in *Kit^wsh−/−^* mice (Figure 7(c) and (d)) suggesting that the doses of compound 48/80 used in this study exerted its effects though mast cell-mediated mechanisms.

### Compound 48/80-mediated mast cell degranulation is not TLR4 mediated

**Figure 8.**
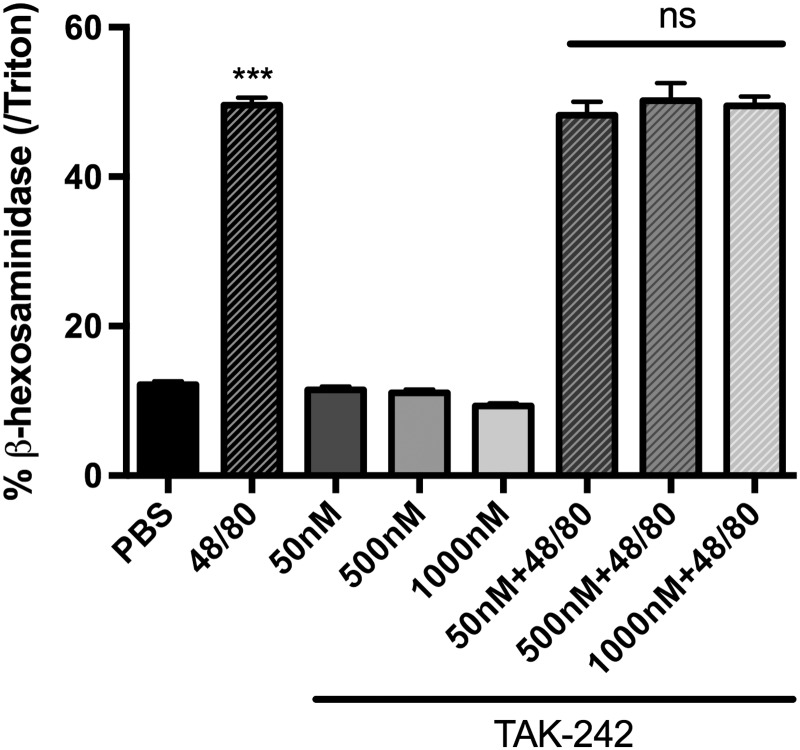
Compound 48/80-induced β-hexosaminidase release from untreated mast cells were no different from those of TAK-242 pre-treated mast cells, suggesting that TLR4 does not influence 48/80-mediated mast cell degranulation. Bar graph represents percentage of β-hexosaminidase release from murine mast cells following treatments with different concentrations of TAK-242 (50 nM, 500 nM, and 1000 nM) 1 h prior to compound 48/80 (10 μg/ml), N = 8. ***P < 0.001 as compared to PBS. ns: non-significant as compared to compound 48/80 treated group; PBS: phosphate-buffered saline.

The presence of TLR4 receptors on mast cells has been reported.^27^ It was therefore important to determine whether compound 48/80-induced mast cell degranulation was TLR4 mediated. To study this issue, we treated the murine mast cells with compound 48/80 (10 μg/mL) for 20 min at 37°C. As expected, compound 48/80 induced degranulation of mast cells evidenced by β-hexosaminidase release compared to the control. We then examined the effect of the TLR4 antagonist, TAK-242 on compound 48/80 induced mast cell degranulation. For this, mast cells were pre-treated with different concentrations of TAK-242 (50 nM, 500 nM, and 1000 nM) on murine mast cells for 1 h prior to treatment with compound 48/80. These doses were chosen on the basis of our recently published study showing an IC 50 = 93 nM for TAK-242.^26^ Compound 48/80-induced β-hexosaminidase release from untreated mast cells was no different than that observed in TAK-242 pre-treated mast cells (Figure 8), suggesting that TLR4 does not influence 48/80-mediated mast cell degranulation. Since the role of TLR4 signaling was crucial in compound 48/80-induced behavior and neuronal activation only in males, the murine mast cell culture in this study was isolated exclusively from male mice.

## Discussion

In this study, we provide a direct evidence of involvement of TLR4 in migraine using the mast cell-mediated model of trigeminal activation. In these studies, manipulations of TLR4 signaling were accomplished by (i) mutation of TLR4 expression, (ii) genetic disruption of signaling pathways downstream to TLR4, and (iii) pharmacological antagonism of TLR4 signaling. These manipulations of TLR4 function had no effect upon normal behavioral function, baseline light-dark preference, but uniformly produced a significant sex-dependent reversal of 48/80-induced photophobia and TNC (n. caudalis) p-ERK activation. We believe these convergent observations each provide substantial support for the role of TLR4 signaling in the male related to the migraine phenotype. Consistent with the behavioral and neuronal effects observed in *Tlr4*^−/−^ male mice, pharmacological inhibition of TLR4 function using TAK-242 antagonist significantly attenuated the 48/80-induced photophobia and p-ERK activation. In the following sections, we will review several issues pertinent to the interpretation of these migraine data sets.

### Compound 48/80-induced light aversion: A surrogate maker for migraine?

Hypersensitivity to sensory stimuli, particularly light (e.g., photophobia), is one of the major symptoms associated with migraine in humans. Over 80% of migraineurs report severe sensitivity to light during and between migraine attack.^2^ In various rodent models of migraine, such sensitivity to light has been widely reported.^28–31^ In this study, we demonstrate photophobia after i.p. administration of a sub-anaphylactic dose of compound 48/80. In WT mice, i.p. compound 48/80 induced light aversive behavior starting by 15 min that lasted up to 2 h with a reversal by 4 h in males and 2 h in females. An important issue is to what degree is photophobia induced by mast cell degranulation a measure of a migraine? Systemic compound 48/80 can cause allergic conjunctivitis, sickness behavior, or may also have effects on central nervous system mast cells. However, the light aversion seen in this study may not be a result of conjunctivitis since pre-treatment with sumatriptan, a specific anti-migraine drug, was able to block 48/80-induced light aversions. Furthermore, it has been argued that sickness behavior could cause anxiety provoking stimulus conditions that may be associated with photophobia. Sumatriptan, however, has no anti-anxiety action and in fact can result in increased anxiety indices,^28^ which we confirmed in our studies. In contrast, we here show convincingly that pre-treatment with a migraine therapeutic sumatriptan significantly reduces light aversion in both males and females, supporting the assertion that whatever else is indicated by the presence photophobia, in the present model, anxiety is not likely to be a contributing variable to this behavior.

### Compound 48/80-induced trigeminal activation

Pain signaling to the brainstem from the meninges by trigeminal vascular afferents has been proposed to be a key event in the pathophysiology of migraine.^29–31^ Intracranial degranulation of mast cells in the dura leads to the release of a variety of pro-inflammatory mediators which generates a state of “sterile inflammation,”^3,4,9^ thereby sensitizing and activating the trigeminal nociceptors involved in the migraine pain pathway.^32,33^ Studies have documented that systemic administration of sub-anaphylactic doses of compound 48/80 causes dural mast cell degranulation and plasma extravasation that promotes persistent activation and sensitization of meningeal afferents and second-order neurons in the meningeal afferent pathway (n. caudalis neurons).^8–10^ Degranulation of mast cells leads to a robust release of a variety of mediators, many of which have been implicated in mast cell-mediated sensitization and activation of meningeal nociceptors.^34^ The specific mast cell mediator(s) involved in these behavioral effects is yet to be determined.

Apart from behavior, we employed an index of neuronal activation in the TNC of these mice through quantification of p-ERK (+) neurons. In accordance with the presence of light aversion, we noted a significant increase in p-ERK (+) cells in the TNC following systemic compound 48/80, both in males and in females. Consistent with effects upon behavior, this activation was attenuated following pre-treatment with sumatriptan. Comparable findings of TNC neuronal activation, using c-fos as a marker, have been reported following i.p compound 48/80.^8^

### Role of mast cells in compound 48/80 actions

The present studies demonstrated that 48/80 resulted in a robust photophobia. The effects of 48/80 are considered to be mediated by mast cell degranulation.^35^ The present work sought to support that association by demonstrating that the 48/80 effects were prevented by cromolyn, a so called mast cell stabilizer.^36^ We note that there is some controversy as to whether 48/80 in fact acts on murine mast cells (in culture) in a cromolyn-sensitive fashion.^37^ However, more recent work by Chakraborty et al. in murine mast cell culture clearly shows a robust concentration-dependent effect of cromolyn in blocking 48/80 evoked calcium influx and degranulation (as measured by release of histamine and ß-hexaminidase) and prevented 48/80-induced shock.^38^ Furthermore, though indirect, it has been reported that the effects of intracerebral 48/80 in mice (leading to brain mast cell degranulation) on behavior were blocked by intracranial delivery of cromolyn, suggesting an action on murine brain mast cells.^39^

With regard to the hypothesized role of mast cells, two additional issues must be considered. First, an important control is whether TLR4, mediating a component of the photophobia, itself mediates 48/80-induced mast cell degranulation. One recent study noted that mast cells express TLR4.^40^ We, however, exclude this possibility in our *in vitro* work with mast cells, showing that compound 48/80-induced degranulation TLR4 antagonism (TAK-242) at drug concentrations blocked LPS-induced macrophage activation.^26^

A second important point is whether mast cells are required for this effect of 48/80. A recent study reported that 48/80, apart from mast cell degranulation, may also have a direct stimulatory effect on primary afferent dorsal root ganglion neurons.^41^ However, we directly addressed this issue here by noting that at the dose of compound 48/80 required to produce p-ERK activation in the TNC, no such neuronal activation was observed in the mast cell deficient *Kit^wsh−/−^* mice. These observations suggest that while 48/80 might have potentially a direct impact upon afferent activity, they confirm that the 48/80 effects observed in this study are mast cell mediated.

### TLR4-mediated signaling pathway

Among the TLRs, TLR4 utilizes both MyD88 and TRIF adapter protein pathways for downstream signaling. Activation of MyD88 can activate nuclear factor-kB (NF-kB) and promote the release of pro-inflammatory cytokines, whereas signaling through TRIF can result in the production of IFN-β and delayed activation of NF-kB to release pro-inflammatory cytokines which are considered to respectively act neuraxially to facilitate and suppress nociceptive processing.^42^ In this study, deletion of these specific adapter proteins showed that TLR4 signaling in this model was MyD88 dependent and TRIF independent in males. At this point, it is difficult to infer the cellular network through which this TLR4-MyD88 signaling is mediated. Functional TLR4 and MyD88 signaling is present in microglia, astrocytes, and neurons and plays a prominent role in the initiation of pain states at the level of the first- and second-order sensory neurons.^43–45^ Importantly, most migraine research targets the biology of sensory neurons, but given the potential role of neuro-inflammatory signaling, non-neuronal cell types such as astrocytes and microglia must also be examined. This provides opportunities for further investigation in this model.

### Is the role of TLR4 activation male specific

Consistent with little effect seen in Tlr4^−/−^ females neither MyD88 nor TRIF disruption showed any effect on compound 48/80-induced light aversive behavior in females. Similarly, double deficiency of adaptor proteins did not prevent compound 48/80-induced light avoidance. These findings suggest that TLR4 in this model may not play a role in compound 48/80-mediated effects in females and that a different mechanism may exist in females as compared to males. Studies by Sorge et al. suggest a sex differential involvement of innate and adaptive immune system. Involvement of spinal TLR4 in inducing mechanical allodynia is suggested to be male specific, whereas females preferentially used adaptive immune cells (T lymphocytes).^44^ A recent study reported significant increase of T-cells in the dura of females following 24 h stress implicating its role in sex specific migraine pathogenesis.^7^

One may consider this to be a limitation of the present study; however, we deem this phenomenon to be intriguing that males and females may utilize different pathological mechanisms for processing pain in migraine. We believe these are highly provocative experiments that have hitherto not been accomplished.

### Toll-like receptor 4 and its role in migraine

The role of functional TLR4s in neuronal and non-neuronal cell types has been gaining attention in pain models such as in K/BxN serum transfer-induced arthritis model and in late phase allodynia following formalin^26^ as well as in other pain disorders related to nerve injury.^42,44,45^ The role of neuro-immune interactions and subsequent release of pro-inflammatory cytokines have been shown to contribute to the initiation of facilitated pain states, that may occur in the trigeminovascular system believed to underlie the migraine phenotype. Further work is required to define the relevance of these effects in humans, but an interesting parallel of this role of TLR4 signaling in migraine is the report that naloxone was reported to be effective in treating acute migraine attacks.^46–48^ While this naloxone effect was reasonably interpreted as reflecting the potential role of endogenous opioids, it has in fact more recently been reported that both the opioid active (−) naloxone and the non-opioid active isomer (+) naloxone can block TLR4 receptor function.^49,50^ This thus raises the alternate interpretation that these human clinical results support the role proposed in our work of TLR4 in the manifestation of the migraine phenotype.

## Supplemental Material

Supplemental material for Role of Toll-like receptor 4 signaling in mast cell-mediated migraine pain pathway Click here for additional data file. Supplemental Material for Role of Toll-like receptor 4 signaling in mast cell-mediated migraine pain pathway by Roshni Ramachandran, Zhenping Wang, Christian Saavedra, Anna DiNardo, Maripat Corr, Susan B Powell and Tony L Yaksh in Molecular Pain
