# Supplementary material for: Role of Toll-like receptor 4 signaling in mast cell-mediated migraine pain pathway
Source: Mol Pain. 2019 Aug 8;15:1744806919867842. doi: 10.1177/1744806919867842 (PMC6688145; doi:10.1177/1744806919867842)
Supplement: Supplemental material for Role of Toll-like receptor 4 signaling in mast cell-mediated migraine pain pathway [file Supplemental_Material.pdf]

1) Statistical analyses for figure 1:

	Male		Female	
Bonferroni's multiple comparisons test	Summary	Adjusted P Value	Summary	Adjusted P Value
**Saline, i.p - Cmpd 48/80**				
Pre	ns	>0.9999	ns	>0.9999
15 min	**	0.0014	**	0.0041
1 hr	***	0.0008	*	0.0491
2 hr	ns	0.077	ns	>0.9999
4 hr	**	0.0091	ns	>0.9999
**Suma + Cmpd 48/80 - Saline + Cmpd 48/80**				
Pre	ns	>0.9999	ns	>0.9999
15 min	**	0.0093	**	0.0028
1 hr	**	0.0062	ns	>0.9999
2 hr	ns	0.2493	ns	>0.9999
4 hr	ns	>0.9999	ns	>0.9999

2) Statistical analyses for figure 2:

Dunnett's multiple comparisons test	Center enteries		Duration in the center		Distance moved	
	Summary	Adjusted P Value	Summary	Adjusted P Value	Summary	Adjusted P Value
0-5						
Vehicle control vs. Cmpd 48/80 (2 mg/kg) i.p	ns	0.0888	ns	0.7602	ns	0.2181
Vehicle control vs. Suma + cmpd 48/80	***	0.0001	ns	0.7443	****	<0.0001
5-10						
Vehicle control vs. Cmpd 48/80 (2 mg/kg) i.p	ns	0.3762	ns	0.6667	ns	0.1567
Vehicle control vs. Suma + cmpd 48/80	****	<0.0001	ns	0.0644	****	<0.0001
10-15						

Vehicle control vs. Cmpd 48/80 (2 mg/kg) i.p	ns	0.6988	ns	0.6897	ns	0.6959
Vehicle control vs. Suma + cmpd 48/80	****	<0.0001	*	0.0282	****	<0.0001
15-20						
Vehicle control vs. Cmpd 48/80 (2 mg/kg) i.p	ns	0.3374	ns	0.077	ns	0.5261
Vehicle control vs. Suma + cmpd 48/80	****	<0.0001	***	0.0004	****	<0.0001
20-25						
Vehicle control vs. Cmpd 48/80 (2 mg/kg) i.p	ns	0.8565	ns	0.1656	ns	0.7802
Vehicle control vs. Suma + cmpd 48/80	***	0.0005	**	0.0016	***	0.0002
25-30						
Vehicle control vs. Cmpd 48/80 (2 mg/kg) i.p	ns	0.7697	*	0.0398	ns	0.9115
Vehicle control vs. Suma + cmpd 48/80	***	0.0002	****	<0.0001	***	0.0009

3) Statistics analyses for figure 3 and 4:

Dunnett's multiple comparisons test	Male		Female	
	Summary	Adjusted P Value	Summary	Adjusted P Value
Pre				
/<br>Saline + 48/80 vs. *Tlr4*−/− + Cmpd 48/80	ns	0.995	ns	0.9727
/<br>Saline + 48/80 vs. *Myd88*−/− + 48/80	ns	0.409	ns	0.9986
/<br>Saline + 48/80 vs. *Trif*−/− + Cmpd 4880	ns	0.9556	ns	0.9715
Saline + 48/80 vs. *Ticam1*^{lps2}/*Myd88*$^{/}$ + Cmpd 4880	ns	0.9102	ns	0.9928
15 min				
/<br>Saline + 48/80 vs. *Trif*−/− + Cmpd 48/80	**	0.0057	ns	0.9967
/<br>Saline + 48/80 vs. *Myd88*−/− + 48/80	*	0.0114	ns	0.5157
/<br>Saline + 48/80 vs. *Trif*−/− + Cmpd 4880	ns	0.8392	ns	0.6992

Saline + 48/80 vs. *Ticam1*^{lps2}/*Myd88*$^{/}$ + Cmpd 4880	ns	0.3245	ns	0.1899
1 hr				
Saline + 48/80 vs. *Tlr4*−− + Cmpd 48/80 /	*	0.0107	ns	0.9944
Saline + 48/80 vs. *Myd88*−− + 48/80 /	***	0.0001	ns	0.8459
Saline + 48/80 vs. *Trif*−− + Cmpd 4880 /	ns	0.9369	ns	0.5056
Saline + 48/80 vs. *Ticam1*^{lps2}/*Myd88*$^{/}$ + Cmpd 4880	*	0.044	ns	0.9986
2 hr				
Saline + 48/80 vs. *Tlr4*−− + Cmpd 48/80 /	ns	0.0943	ns	0.1907
Saline + 48/80 vs. *Myd88*−− + 48/80 /	*	0.0344	ns	0.999
Saline + 48/80 vs. *Trif*−− + Cmpd 4880 /	ns	0.9806	ns	0.458
Saline + 48/80 vs. *Ticam1*^{lps2}/*Myd88*$^{/}$ + Cmpd 4880	ns	0.5671	ns	0.6508
4 hr				
Saline + 48/80 vs. *Tlr4*−− + Cmpd 48/80 /	ns	0.2538	ns	0.6707
Saline + 48/80 vs. *Myd88*−− + 48/80 /	ns	0.1392	ns	0.0829
Saline + 48/80 vs. *Trif*−− + Cmpd 4880 /	ns	0.6482	ns	0.984
Saline + 48/80 vs. *Ticam1*^{lps2}/*Myd88*$^{/}$ + Cmpd 4880	ns	0.8591	ns	0.8655
**Bonferroni's multiple comparisons test**				
Saline + Cmpd 48/80 - TAK-242 + Cmpd 48/80				
Pre	ns	>0.9999	ns	>0.9999
15 min	ns	0.9338	ns	>0.9999
1 hr	*	0.0426	ns	>0.9999
2 hr	ns	0.1988	ns	0.123
4 hr	ns	>0.9999	ns	0.7516

4) Statistical analyses for figure 5 and 6:

**Dunnett's multiple comparisons test**	**Male**		**Female**	
**p-ERK line graph**	**Summary**	**Adjusted P Value**	**Summary**	**Adjusted P Value**
Row 3				
Cmpd 48/80 (2 mg/kg) i.p vs. Vehicle	**	0.0095	ns	0.2686

Cmpd 48/80 (2 mg/kg) i.p vs. Suma + cmpd 48/80	ns	0.1662	ns	0.132
Cmpd 48/80 (2 mg/kg) i.p vs. TLR4 KO + Cmpd 48/80	ns	0.1073	ns	0.5969
Cmpd 48/80 (2 mg/kg) i.p vs. TAK-242 + Cmpd 48/80	ns	0.0949	ns	0.6811
Row 4				
Cmpd 48/80 (2 mg/kg) i.p vs. Vehicle	****	0.0001	****	0.0001
Cmpd 48/80 (2 mg/kg) i.p vs. Suma + cmpd 48/80	*	0.0243	*	0.0144
Cmpd 48/80 (2 mg/kg) i.p vs. TLR4 KO + Cmpd 48/80	*	0.0131	ns	0.1697
Cmpd 48/80 (2 mg/kg) i.p vs. TAK-242 + Cmpd 48/80	***	0.0004	ns	0.9389
Row 5				
Cmpd 48/80 (2 mg/kg) i.p vs. Vehicle	****	0.0001	****	0.0001
Cmpd 48/80 (2 mg/kg) i.p vs. Suma + cmpd 48/80	***	0.0003	ns	0.0592
Cmpd 48/80 (2 mg/kg) i.p vs. TLR4 KO + Cmpd 48/80	*	0.0104	ns	0.998
Cmpd 48/80 (2 mg/kg) i.p vs. TAK-242 + Cmpd 48/80	***	0.0003	ns	0.9999
Row 6				
Cmpd 48/80 (2 mg/kg) i.p vs. Vehicle	***	0.0005	**	0.003
Cmpd 48/80 (2 mg/kg) i.p vs. Suma + cmpd 48/80	*	0.0458	ns	0.9612
Cmpd 48/80 (2 mg/kg) i.p vs. TLR4 KO + Cmpd 48/80	ns	0.1518	ns	0.9999
Cmpd 48/80 (2 mg/kg) i.p vs. TAK-242 + Cmpd 48/80	ns	0.0514	ns	0.8517
**p-ERK bar graph**<br>**Sidak's multiple comparisons test**				
Saline vs. 48/80	***	0.0002	**	0.0013
48/80 vs. Suma + 48/80	*	0.0137	*	0.037
48/80 vs. Tlr4 $^{-/-}$ + 48/80	*	0.0343	ns	0.7693
48/80 vs. TAK-242 + 48/80	**	0.0031	ns	0.9986

5) Statistical analyses for figure 7:

Dunnett's multiple comparisons test	Male	
	Summary	Adjusted P Value
Pre		
Saline + 48/80 vs. Cromolyn + 48/80	ns	0.8803
Saline + 48/80 vs. Saline, i.p	ns	0.7052
15 min		
Saline + 48/80 vs. Cromolyn + 48/80	*	0.0268
Saline + 48/80 vs. Saline, i.p	**	0.0097
1 hr		
Saline + 48/80 vs. Cromolyn + 48/80	***	0.0005
Saline + 48/80 vs. Saline, i.p	*	0.0241
2 hr		
Saline + 48/80 vs. Cromolyn + 48/80	*	0.0227
Saline + 48/80 vs. Saline, i.p	ns	0.2292
4 hr		
Saline + 48/80 vs. Cromolyn + 48/80	ns	0.5282
Saline + 48/80 vs. Saline, i.p	ns	0.2225
**Sidak's multiple comparisons test**		
WT + Cmpd 48/80 - $Kit^{wsh-/-}$ + Cmpd 48/80		
Row 3	ns	0.0528
Row 4	**	0.0019
Row 5	**	0.0013
Row 6	*	0.0357
